# Road pavement upgrade scheduling accounting for minimizing congestion

**DOI:** 10.1038/s41598-023-40945-5

**Published:** 2023-09-16

**Authors:** Bhagya Rupasinghe, Mark Wallace, Graeme Gange, Ilankaikone Senthooran

**Affiliations:** 1 Monash University, Wellington Road, Clayton, VIC 3800 Australia; 2 ARC Training Centre in Optimisation Technologies, Integrated Methodologies, and Applications (OPTIMA), The University of Melbourne, Melbourne Connect, 700 Swanston St, Melbourne, VIC 3010 Australia

**Keywords:** Computer science, Information technology, Software

## Abstract

Road pavement maintenance and upgrades (RPU) need to be scheduled in a way that minimises congestion across the road network and matches the schedule with the availability of equipment and skilled labourers to ensure that they are completed on time. RPU should not introduce unbearable congestion around blocked lanes or roads, and increased traffic due to ongoing road upgrades needs to be transferred to alternative routes. In situations where multiple upgrades are planned together, the scheduled road closures should be coordinated to not block alternative routes. While extensive studies have been conducted separately on scheduling and routing, limited research has linked these processes together, and only in restricted scenarios. In this paper, we investigate how to minimise the disruption of a set of road maintenance tasks based on their interaction with other tasks. We propose a new approach for scheduling a set of road maintenance tasks, some at the same time and others at different times, to minimise overall disruption. The proposed approach has two phases: (1) identify the local area affected by the RPU, and (2) determine which upgrades can be planned together to minimise congestion. The comparison of the results shows how the novel optimized approach identifies a better schedule to minimise congestion.

## Introduction

Road networks play a significant role in supporting transportation efficiency, and consequently, the economy. A damaged road network can hinder daily commuters, affecting their productivity and ultimately impacting the country’s overall economy. Therefore, road pavement maintenance is crucial in maintaining a productive ecosystem for a prosperous society. Some maintenance tasks require immediate attention, such as repairing road damage caused by severe weather or accidents, while others may be less urgent but still require maintenance or upgrades in the future. Each of these tasks requires different resources depending on their needs. Planning is essential to allocate and schedule resources optimally, while maintaining a high-quality road network.

**Figure 1:**
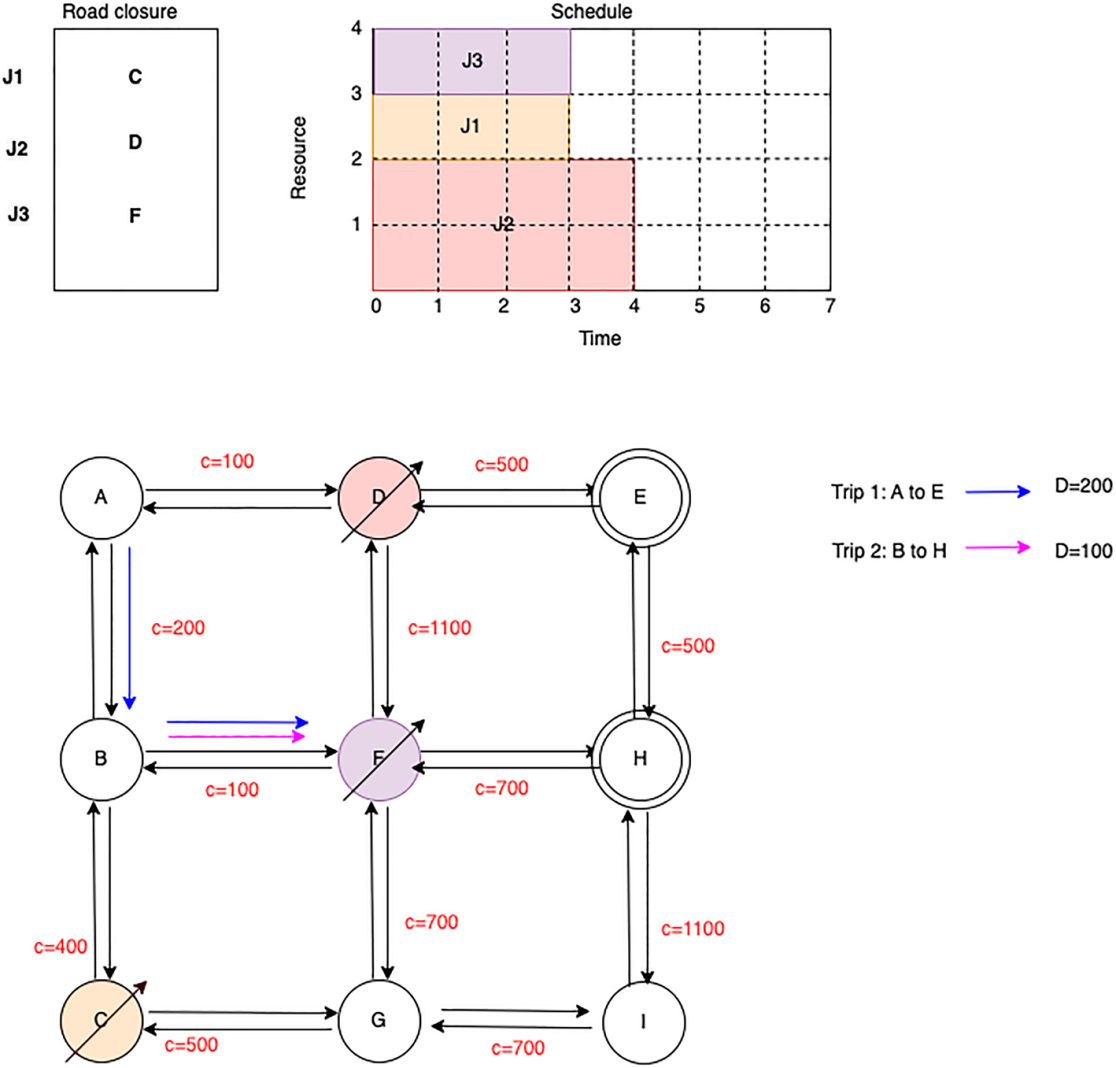
Scheduling RPU considering availability of resources.

**Figure 2:**
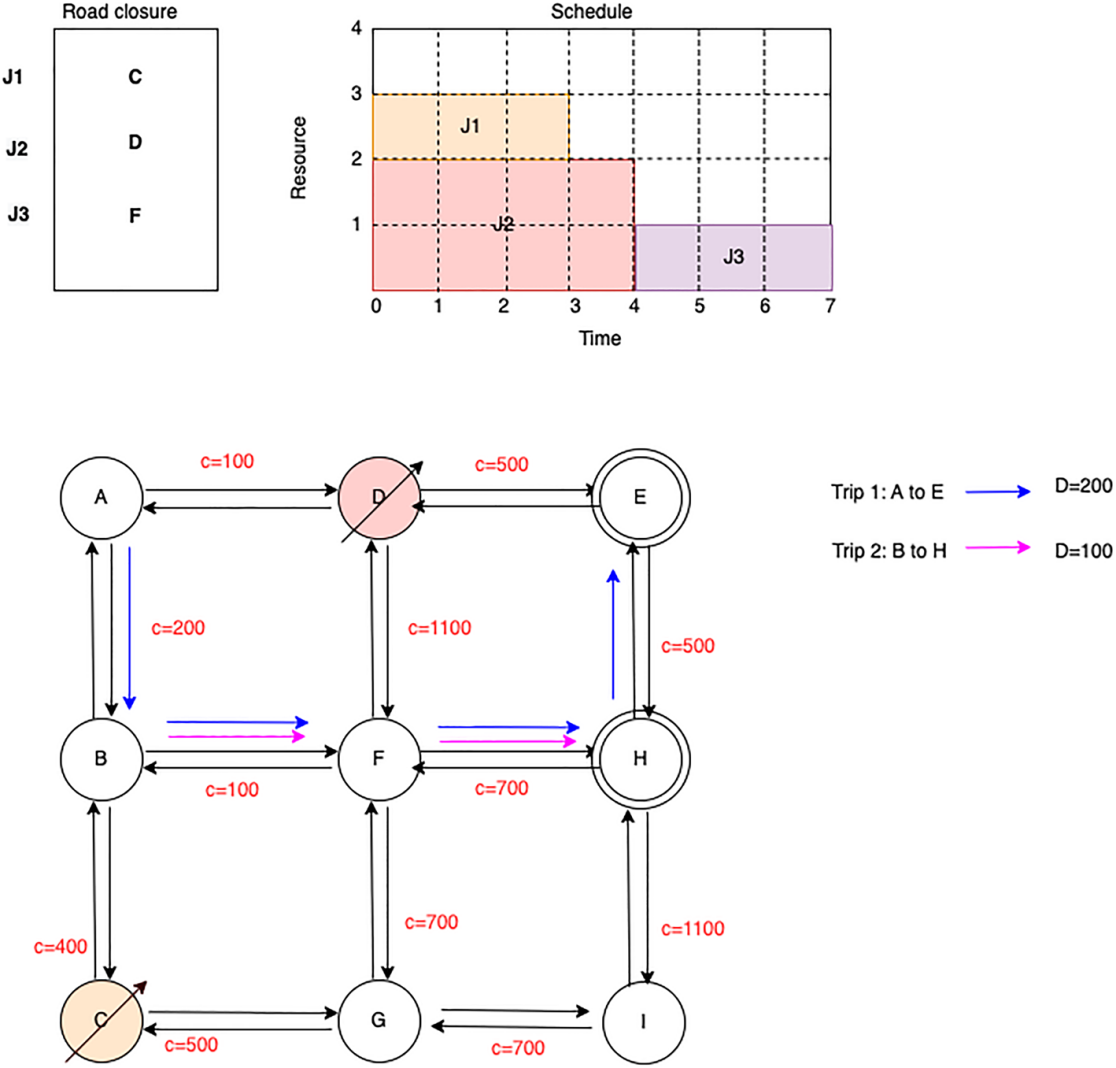
Scheduling RPU considering their effect on road network.

It is possible to schedule multiple road upgrades on the same day, taking into account their urgency and the availability of resources. The Fig. 1 illustrates a road network and the proposed road pavement maintenance and upgrade schedule (RUS). Spanning from A through I, the network comprises nine nodes representing road junctions, each with incoming and outgoing road segments/edges. The program encompasses three upgrades, each requiring the closure of three distinct nodes: C, D, and F. J2, J3, and upgrade task J1 are all scheduled to run simultaneously.

Two separate trips are needed to travel from point A to E and from point B to H, with demands of 200 and 100, respectively. Upon inspection of the diagram, it can be inferred that scheduling all three jobs on the same day or at the same time, while only considering resources, would result in the network being inaccessible to traffic due to the blockage of alternative routes. This would effectively impede both trips from reaching their respective destinations on the Fig. 1.

However, if the road upgrades are scheduled while also taking into account their impact on the corresponding road segments, the traffic can be directed to their respective destinations without obstruction. As demonstrated on the Fig. 2, a two-stage approach can be employed to plan the road upgrades without blocking alternative routes. The remarkable interaction between the maintenance sites that were planned together highlights the importance of considering the interdependence between maintenance sites when scheduling them. These findings underscore the significance of accounting for the interactions between maintenance sites during the scheduling process.

Therefore, it is important to consider how to schedule urgent maintenance tasks to minimize their impact on the community’s mobility. This could involve scheduling tasks individually or simultaneously, but the total disruption caused by simultaneous scheduling may be greater or less than that caused by scheduling them at different times. Addressing these issues requires finding the best combination of road upgrade tasks to schedule, while reducing congestion by choosing a set of upgrades that have possible detours or traffic reroutings. The diverted additional demand towards alternative routes should be designed to avoid queue generation due to exceeding capacity on the existing road segments. Furthermore, when multiple upgrades are planned together, the scheduled road closures should be coordinated so as not to block alternative routes.

The present study proposes an approach for scheduling a set of road maintenance tasks, some to be executed simultaneously and others at different times, in order to minimize overall disruption in large networks, such as those in Melbourne, Australia and Chicago, USA. The algorithm has the capability to identify jobs that have the most significant impact when planned together. It then devises maintenance work plans that minimize traffic delays and congestion. By utilizing this algorithm, transport authorities can allocate funds for road repairs in the most efficient manner. This approach enables the effective utilization of manpower, materials, and machinery, thereby reducing costs and yielding improved maintenance outcomes. Consequently, travel times for both commuters and commercial vehicles are decreased, enhancing the overall effectiveness of the road network. Our proposed method involves the development of a naive approach for scheduling road upgrades, which considers all possible combinations of the given road maintenance tasks and selects the best combination that leads to minimal disruption. We then optimize this naive approach using a local disruption-based approximation.

The first section of the paper will provide an overview of the background and the development of the naive approach. Subsequently, the results section will present the outcomes of the suggested algorithms. The discussion section will then compare and analyze the findings from all algorithms. Finally, the paper will conclude by explaining the methodology employed in this study.

## Related work

Scheduling pavement upgrades necessitates the implementation of a mechanism for modeling the impact of closed road segments on traffic flows. In particular, traffic traveling towards the site of road closure must be diverted to alternative routes without causing congestion on those routes. Within the framework of traffic assignment, road users make choices between competing routes, which may include the shortest but congested routes, overloaded as a result of the maintenance work. Alternatively, longer routes may be less congested and offer a faster travel experience. Travelers generally avoid congested routes and opt for faster alternatives such that every traveller uses the route that makes their journey quickest.

Multiple upgrades along the alternative routes connecting major centres should not be executed simultaneously. Still, on the other hand, multiple upgrades along a single route should preferably be synchronised. The proposed solution will consider multiple upgrade scheduling without blocking alternative routes. Addressing these limitations requires finding the best alternative path to minimise travel duration while reducing congestion by offering different detours. The diverted additional demand towards alternative routes should be designed to avoid queue generation due to exceeding capacity on the road segments.

Transportation network-based decisions such as travel time in alternative roads mostly consider the immediate environment. However, the upgrade site’s blockage can cause a ripple effect through the whole road network instead of a closed area for the road pavement upgrades. The problem needs to consider the entire road network as the affected site when deriving the solution. The best deviation approach is considered to be the shortest path from the maintenance site to the destination. Frequent selection of the shortest path may lead to congestion, where delay makes it a non-optimal route. The ripple effect from road closure on neighbouring streets was evaluated by^1^. The study aimed to develop a predictive model that could estimate the traffic flow rate in a specific road segment based on the speed of vehicles on nearby road segments. The authors used data from a large urban road network in Jakarta, Indonesia, and developed a multiple regression model to predict traffic flow. The model used a set of independent variables, including the average speed of neighboring roads and time of day, to predict the dependent variable, which was the traffic flow rate. The results showed that the model was able to accurately predict the traffic flow rate with a high degree of accuracy, indicating the effectiveness of the proposed approach. The study demonstrated the potential of using neighboring road data to predict traffic flow rates, which could be valuable for traffic management and planning in urban road networks.

Ma et al.^2^ suggested a dynamic programming approach to minimize the delay caused by the road maintenance scheme. The solution recommends finding out the best combination of road segments to maintain simultaneously to reduce hindrance. The simultaneous updates depend on the availability of machinery, labours and budget. Systems use traffic managers to decide the number of links to upgrade simultaneously based on their maintenance schedule and the available budget. According to the authors, getting input from traffic managers for the number of links to plan simultaneously gives the managers a chance to identify the best upgrade schedule by changing input parameters.

Genetic algorithm (GA) is a type of optimization algorithm inspired by the process of natural selection. It is used to solve problems that involve finding the optimal solution from a large set of potential solutions.The GA algorithm can be effective in finding optimal solutions in complex search spaces, particularly when the problem involves many variables or constraints. However, the GA can be computationally expensive, particularly when the population size is large or the objective function is complex. Also, on any particular problem there is no way to know how good the result produced by a GA is (after any amount of computation time). For example, setting some GA parameter differently (or using a different optimisation technique) might have quickly produced a much better result.

Chakroborty et al.^3^ suggested a GA to find optimal traffic assignments for the Singapore road network. The suggested GA uses radius-based preconditions to generate the initial population by identifying a part of the network within the traffic condition’s affected radius. The authors emphasize the importance of initial population detection in reducing error criterion.

Due to the extensive duration of most pavement upgrade sites, hourly closure site solutions, as presented in^4^, are not practical since they do not account for the time required for multiple sites. In response, day-to-day traffic assignment under work zone scheduling, as proposed by^5^, has emerged as a more feasible research method. Yang et al.^6^ presents a day-based work zone scheduling model for urban road networks, considering day-to-day traffic variations. The objective of this model is to minimize the total travel time of road users while ensuring that the work zone activities are completed within a specified time frame. However, it should be noted that the developed solution is theoretical and lacks practical validation with data.

Infrastructure management is an important aspect of traffic assignment. During road upgrades, traffic management typically provides alternative routes for drivers. However, these routes may not always be the most efficient and knowledgeable local drivers may choose better pathways. In contrast, non-local drivers may rely on the detours provided by road management authorities, even if they are longer than necessary. This can result in congestion on detours, especially if these routes are also chosen by local drivers. To address this issue, ter Huerne et al.^7^ proposed an infrastructure management system that balances total cost and traffic hindrance. They presented an alternative traffic assignment definition that considers more factors than just speed and flow, resulting in a theoretical framework for managing infrastructure.

Abdelmohsen and El-Rayes^8^ proposed a multi-objective genetic algorithm to minimize traffic delays and the probability of crashes in work zones. The authors considered several work zone properties, such as work zone length, speed reduction factors (e.g., redirect signs), and traffic demand, to determine the potential delays caused by work zones.

In Australia, most road upgrades require full road closures, which often result in travellers taking detours instead of encountering lane closures. However, the approach discussed in^8^ does not consider alternative routes. In contrast, the spillback process described in^4^ reduces traffic towards the lane closure, which differs from the suggested rerouting solution.

In order to maintain the safe and effective operation of road networks, pavement management and repair are essential elements of highway infrastructure management. Preventive maintenance (PM) and rehabilitation procedures that are carefully planned can greatly increase the service life of pavements while minimising costs and inconveniences for road users. In order to address the difficulties of optimising pavement maintenance schedules, the numerous optimisation models and approaches put out in recent research.

Lamptey et al.^9^ has shown interest in the creation of genetic algorithms (GAs)-based pavement management systems. By concurrently minimising maintenance costs and maximising pavement condition throughout a planning period, these systems aim to establish an ideal pavement maintenance and rehabilitation approach for highway networks. For pavement engineers, applying multi-objective GAs with powerful search capabilities and constraint handling techniques has been useful^10^.

Gao et al.^11^ proposed a variety of methodologies, including metaheuristic approaches and parametric methods, to address the difficulty of optimising two objectives, i.e., pavement condition improvement and budget utilisation. These approaches seek to offer comprehensive Pareto-optimal solution sets, enabling decision-makers to schedule pavement maintenance and rehabilitation tasks in a way that strikes a good balance.

Hybrid techniques combining genetic algorithms and traffic simulation models have been proposed to reduce travel times and traffic congestion caused by lane closures during pavement restoration activities. These methods take into account both arterial and motorway networks, providing workable strategies for allocating maintenance personnel to numerous project requests^12^.

The traditional approach to bridge maintenance focuses on individual bridges and ignores the interactions between bridges in a network. Wang^13^ suggested a strategy meets this need by giving network-level maintenance first priority. The best time for maintenance is calculated using a predictive maintenance model based on Markov chain deterioration models. Additionally, group maintenance is incorporated into the strategy to cut setup costs, and the scheduling procedure takes budgetary constraints into account. The optimisation strategies utilised to carry out the suggested strategy are also covered in this article, including rolling horizon scheduling and genetic algorithms.

This scientific article^14^ focuses on the problem of highway maintenance scheduling. The authors suggest the minimal makespan strategy (MMS) and the minimal increased travel delay strategy (MITDS) as two alternative methods for planning out highway maintenance tasks. As a mixed integer linear programming model with restrictions on labour and maintenance order, they propose MMS. They create a bi-level programming model for MITDS that consists of an upper level model to minimise longer maintenance windows with increasing traffic delays and a lower level model to simulate traffic evolution. The MMS model is solved by the authors using a simulated annealing approach, and the MITDS model is solved using an augmented Lagrange algorithm.

Overall, the works discussed in this context offer different approaches to minimizing traffic delays during work zones,and the state of the art in scheduling road maintenance optimisation, each with its own strengths and limitations. As far as we are aware, no literature examines how one road upgrade or maintenance site (RUS) affects another. Our research closes this gap by applying the method that is presented in this work, where we analyse the effects of each RUS on the others and create a schedule that minimises congestion while avoiding blocking alternate routes. Future research could explore combining the two approaches or developing new methods that consider both lane closures and detours for work zone scheduling and traffic management.

## Methods

### Combination evaluation model (CEM)

**Figure 3:**
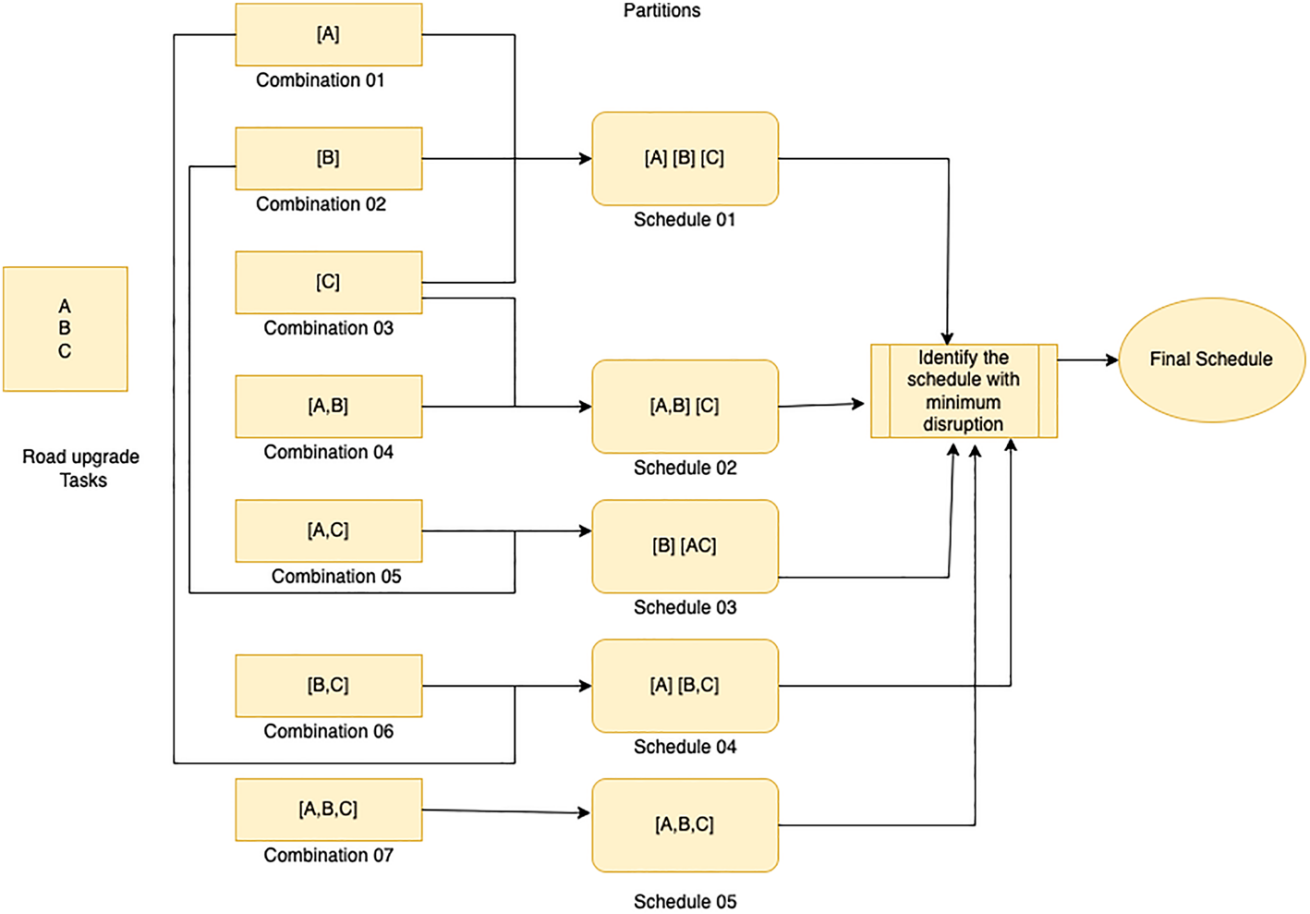
Combination evaluation model.

The proposed solution comprises two steps, wherein road upgrade combinations are first considered naively using the CEM approach, and then an approximation is used to scale the algorithm using local disruption based model LDBM. CEM is a naive approach to scheduling multiple road upgrade tasks, assuming that each maintenance task takes a unit of time (such as a day). When two maintenance tasks occur on the same day, their interaction could either increase or decrease the total disruption to traffic flows. Given a set of maintenance tasks, the scheduling problem is to partition all the maintenance tasks such that all tasks in each component of the partition are performed on the same day. A “combination” is a set of maintenance tasks to be performed on the same day, i.e., a component of the partition. Figure 3 shows an example of three tasks, A,B,C, resulting in seven possible combinations: A, B, C, (A, B), (B, C), (A, C), (A, B, C). Each schedule/partition can be represented by a set of combinations that includes all the tasks. Tasks in the same component of the partition are scheduled simultaneously, while tasks in different components are scheduled at different times, assuming that the component represent different days. In schedule 02 in Fig. 3, tasks A and B are scheduled together on one day, while task C is moved to another day. The disruption is calculated from the increase in travel cost by introducing the combinations of road closure sites to the road network. CEM evaluates the total disruption resulting from each partition and chooses the partition that causes the least disruption to find the optimal schedule.

**Figure 4:**
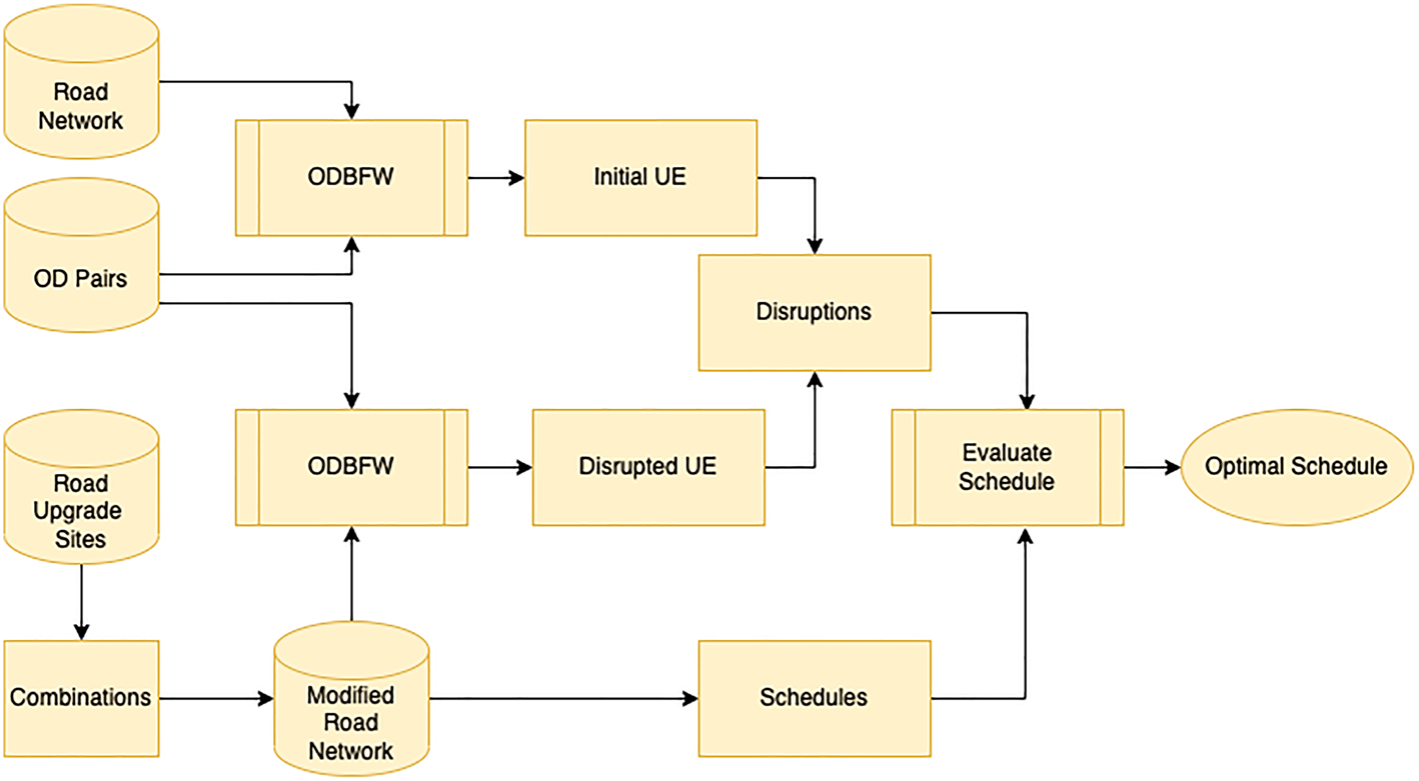
Naive scheduling approach.

The Figure presented in Fig. 4 illustrates how the CEM approach generates the optimal schedule by considering the disruption caused by each partition. The level of disruption is measured as the total travel time for all origin-destination pairs that involve the maintenance tasks, subtracted from the total travel time without any maintenance. The disruption calculation is carried out using the Origin Destination Based Frank Wolfe (ODBFW) algorithm, which is detailed in section “ODBFW”. In brief, the ODBFW algorithm calculates the initial user equilibrium (UE),where the travel demand and route choices of users are in a state of equilibrium. In a user equilibrium, individuals select their routes based on their perceived travel costs, with the objective of minimizing their own travel time or cost. This assumption relies on users possessing perfect information about the network and making rational decisions to optimize their travel. By employing the ODBFW, the initial user equilibrium is determined without considering road upgrade tasks. Next, it calculates the disrupted user equilibrium by introducing road closure sites as blocked links in the network. The resulting disruption caused by each combination is then used by the CEM approach to generate the optimal schedule. The linear constraints-based algorithm then identifies the best schedule by comparing the recorded disruption against the combination of upgrade sites.

Naively, our ODBFW algorithm needs to be executed on each combination. The algorithm runs once for each subset of the maintenance tasks, requiring $2^F-1$ runs for a set of size *F*. Using the example in Fig. 3, there are seven combinations, or $2^3-1$. The road upgrade schedule/partition (S) can be represented as a set of combinations (c), where each combination includes a set of road upgrade tasks (t). Referring to the same example in Fig. 3, [*A*, *B*] and [*C*] are two different combinations used to create schedules [*A*, *B*][*C*], which includes all tasks. The disruptions (D) will vary from one schedule to another when the schedule is deployed to the road network. The road upgrade schedule can be considered as a set of combinations S=1..c where combination includes a set of road upgrade tasks (t). These combinations will have different disruptions (D) by introducing that to the road network. The linear optimization problem was derived to minimize the total disruption generated by the combinations in the selected schedule (1). The selected schedule should include all the road upgrade tasks(t) (2).

Minimize,

1 $$\begin{aligned} Z= \sum _{c \in S} D_{c} \end{aligned}$$

Subject to,

2 $$\begin{aligned} \sum _{c \in S}t \ge 1, \forall t \in c \end{aligned}$$

### Local disruption based model LDBM

This section discusses the approximation algorithm developed to improve the scalability of CEM. Note that in CEM, only a few maintenance sites interact with other maintenance sites, and thus, only these sites need to be evaluated in combination with others. By studying only those sites in combination, the planning of independent sites becomes easier. However, if changes are made globally, the number of partitions increases exponentially with the number of tasks, resulting in many more combinations to evaluate, which makes finding a globally optimal solution challenging. For instance, for four tasks, 15 partitions need to be compared.

The proposed approach to identifying subsets of tasks that cause the same delay, whether scheduled in the same component or not, can significantly reduce the number of partitions that need to be evaluated. For example, consider the case of four tasks where it is assumed that it makes no difference whether task 1 is scheduled in the same component or a different component from tasks 2, 3, and 4. In this case, the disruption due to the partition {1,2},{3,4} is the same as the disruption due to the partitions {1},{2},{3,4} and {2},{1,3,4}. This means that the number of distinct partitions that need to be evaluated is only five instead of 15: [{1},{2},{3},{4} ], [{1},{2},{3,4}],[{1},{2,3},{4}], [{1},{2,4},{3}] and [{1},{2,3,4}].

If we impose a limit on the area around any maintenance task where traffic is disrupted, then the disruptions caused by any two maintenance tasks far enough apart will be independent. Consequently the total disruption caused by these two tasks will be the same, whether they occur at the same time or at different times. Imposing this limit reduces the number of combinations that need to be evaluated. For example, using the previous scenario, if tasks {1,2} and {3,4} can be scheduled independently, only the following combinations need to be evaluated: {1},{2},{1,2},{3},{4}, and {3,4}. The least disruptive combinations from the first two ({{1},{2}} and {1,2}) can be combined with the least disruptive combinations from the second two ({{3},{4}} and {3,4}) in any way that results in the minimum total disruption. Instead of considering all possible combinations, we only need to evaluate combinations of maintenance sites whose disruption ranges overlap. If no local disrupted area around a maintenance site interacts with any other, we can plan the maintenance sites independently without considering combinations.

**Figure 5:**
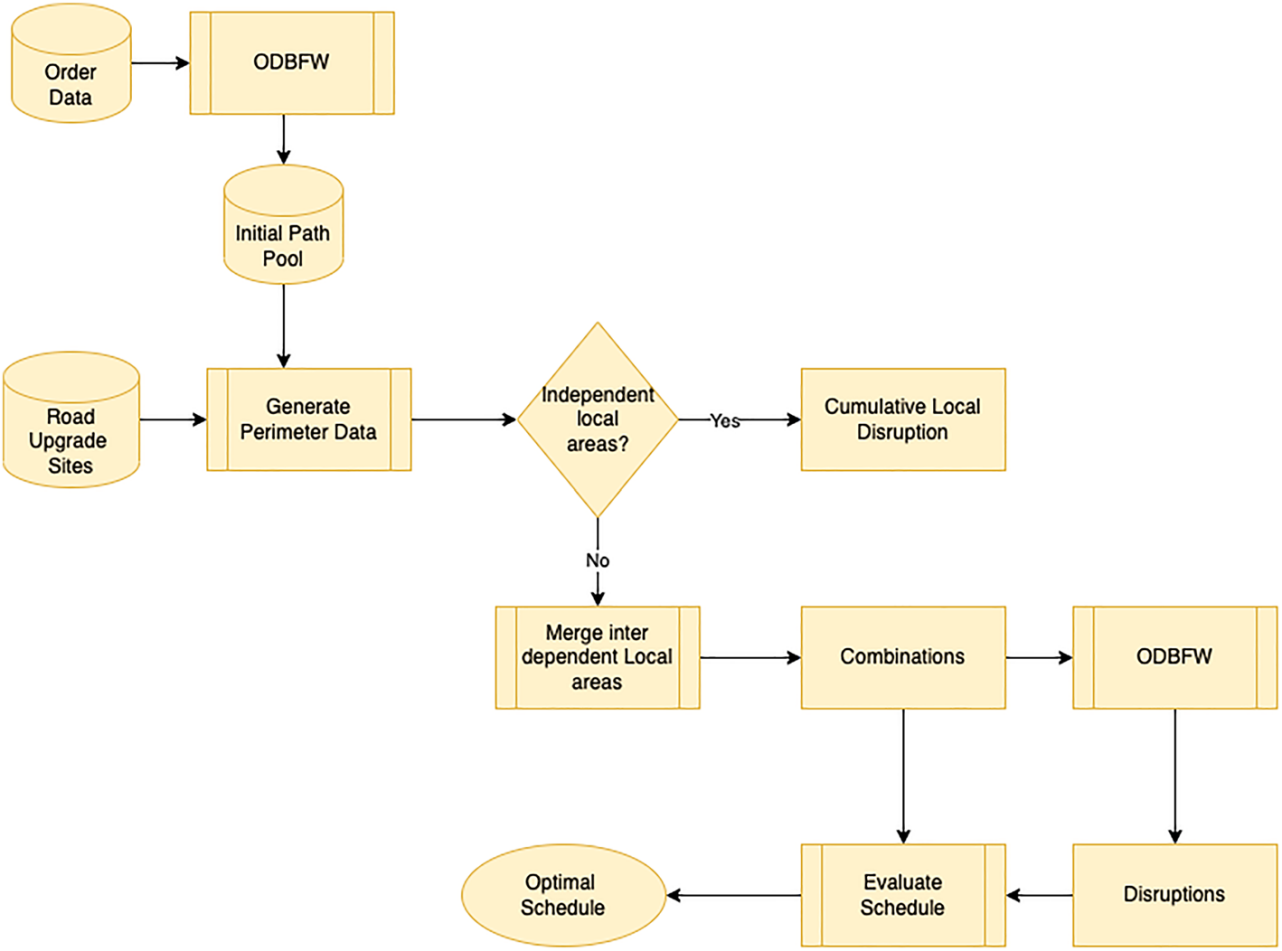
LDBM design.

LDBM requires inputs of OD pairs and a set of road upgrade sites that need to be scheduled, as shown in Fig. 5. The Origin Destination Based Frank Wolfe (*ODBFW*) algorithm discussed in section “ODBFW” is used to determine the best route for all OD pairs, considering the increasing travel time based on increased flow on edges. ODBFW generates initial routes for OD pairs when no upgrade site is planned. Next, the disruption range is determined based on a specified radius, and perimeter data is generated. The algorithm then identifies disruption areas that have a knock-on effect on each other.

As discussed earlier, only a few maintenance sites among all available sites interact with each other. We assume that the considered radius is a distance that affects traffic due to the scheduled road upgrade site. Thus, if two tasks are on roads more than twice this distance apart, the disruption caused by each is considered independent of the other. Only if there are other tasks on roads lying between them could the disruptions have a knock-on effect on each other. As shown in Fig. 5, the generated perimeter data is used to identify independent road upgrade sites. If two local areas contain the same road segments, they have a knock-on effect on each other. If all upgrade sites are independent, they can be introduced to the network one by one to calculate local disruption as each combination will not change the disruption.

In the event of a road upgrade site implementation, the algorithm employed will redirect all origin-destination (OD) pairs entering the designated local area. (1) The ODBFW algorithm applied to the network without maintenance will return all the origin-destination routes. (2) Those routes that cross the perimeter of the designated local area around a maintenance site are selected as those that will be potentially disrupted by the maintenance task. (3) For each such route the subroute (or subroutes) that are within the designated local area are elicited. (4) The start and end points of each such elicited subroute (on the perimeter of the designated area) are recorded as new origins and destinations. (5) The network is modified to exclude both this maintenance site (modelled as a blocked edge in the network) and all edges outside the designated local area. (6) The ODBFW algorithm is applied to the reduced network to optimise all the new origin-destination pairs elicited at (4) above (7). The final disrupted routes for the original OD demands are stitched together from the parts of the original route outside the designated local area, and the disrupted routes within the designated area.

The magnitude of the disruption is computed as the difference between the original edge cost without road upgrade sites in the local area and the edge cost when certain road segments are blocked due to maintenance work. If the local areas operate independently, the cumulative disruption corresponds to the total disruption generated by scheduling all tasks in any given combination, based solely on the available resources.

As previously stated, when local areas share common boundaries, confining the rerouting operations to a single area will prevent the measurement of knock-on effects from the other areas. Under such circumstances, the LDBM amalgamates the local areas and determines the most effective combinations of road closures to be carried out on specific days to minimize the overall disruption within the amalgamated area. The combinations will be introduced to the network one at a time, and their respective disruptions will be calculated using the ODBFW algorithm. The linear constraint-based optimization module expounded upon in section “CEM” will be utilized to ascertain the optimal schedule, taking into account the available combinations and their associated disruptions, with the ultimate aim of minimizing disruption.

### Origin destination based Frank Wolfe (ODBFW)

As elucidated in introduction, road upgrade sites have a direct impact on road traffic conditions, resulting in an increase in travel costs owing to the mounting congestion. Traffic assignment models serve as a solution for assigning traffic flow on a road network. These models take into account traffic flows between origin and destination pairs, assigning traffic to road segments based on the travel time of alternative paths that could potentially carry the traffic. Drivers who make the same origin-destination trip every day modify their routes each day until they find that they cannot improve on their current route. In this case their current route is the quickest, given the congestion on the roads due to all the drivers on their own routes. Given many drivers each with their own origin and destination, the final state where no driver can change to a quicker route is termed the “user equilibrium” state. Interestingly there is another state in which the total travel time for all the drivers is less, but in this case some of the drivers may not be on their quickest route. This is called the “social optimum” state.

The Frank and Wolfe (FW) algorithm is widely used for user equilibrium traffic assignment^15^. Initially, FW was utilized to solve traffic assignment problems by^16,17^. Despite its slow convergence, it gained widespread acceptance due to its simplicity. FW was initially implemented as a link-based algorithm. However, modern practical implementations of FW are path-based, such as route guidance systems. Although FW is an optimization algorithm capable of finding optimal solutions, it has some limitations. As previously mentioned, FW converges slowly and produces paths of significantly lower quality. According to Chen and Jayakrishnan^18^, some paths are unqualified to be included in the path set. In recent years, numerous steps have been taken to enhance the convergence of the FW algorithm. One such effort was undertaken by^19^, who improved FW using an optimized flow update strategy and a column dropping algorithm to remove paths of lower quality.

In traffic assignment, the task of finding the shortest path for each OD pair is computationally expensive, as it requires graph traversal each time the shortest path is needed. To mitigate this issue, the OD-based FW algorithm (ODBFW) stores the shortest path as a tree, wherein the paths from one origin (*s*) to all the destinations (*d*) are kept together to generate the shortest path data (k). Initially, all the demands are assigned using free-flow travel times and the All-or-Nothing (AON) assignment. The path set for source s and destination d, $k^n_{s,d}$, is recorded to perform flow updates, where *n* represents the current iteration. At each iteration, the travel time is modified based on the flow pattern resulting from the previous iteration. Then, the algorithm recalculates the path tree based on the new travel cost. If the shortest path generated from this iteration is different from the previous path set $k^{n-1}{s,d}$, then the new path is added to the path set. If not, the previous path set is retained as the current path set for will be $k^n{s,d}=k^{n-1}_{s,d}$. This process is repeated for all available OD pairs. Based on the new shortest paths the flow will be updated. If the current flow on the link is equal to the previous flow for all the links, then the iteration is stopped. Chen’s ODBFW^18^ is used in LDBM to generate the shortest paths.

### Chen’s improved FW algorithm

Given the graph G(N, A) where N and A represents the nodes and edges, with a set of origins S and destinations D, the traffic assignment problem for fixed demand is stated as,

3 $$\begin{aligned} Z_{min}= \sum _{a \in A} \int \limits _{0}^{x_{a}}t_{a} (w)dw \end{aligned}$$

subject to,

4 $$\begin{aligned}{} & {} \sum _{a \in O(n)} x_{a}^{sd} - \sum _{a' \in I(n)} x_{a'}^{sd} = {\left\{ \begin{array}{ll} q^{sd} &{} if\, n=s\\ -q^{sd} &{} if \,n=d, \quad \forall n \in N, \quad \forall s \in S, \quad \forall d \in D \\ 0 &{} \text {otherwise} \end{array}\right. } \end{aligned}$$

5 $$\begin{aligned}{} & {} \sum _{s,d} x_{a}^{sd} = x_{a}, \quad \forall a \in A \end{aligned}$$

6 $$\begin{aligned}{} & {} x_{a}^{sd} \ge 0, \quad \forall a \in A, \quad \forall s \in S, \quad \forall d \in D \end{aligned}$$

where, I(n), Incoming links towards the node n; O(n), Outgoing links from node n; $$x_{a}^{sd} $$, flow on the link a from OD pair s,d; $$x_{a} $$, total flow on the link a; $$q^{sd} $$, fixed demand for OD pair (s,d); and, $$t_{a}(x_{a})$$ flow dependent link cost function that can differentiable and convex.

In the traffic assignment problem, the solution of the mathematical formulation results in social optimum conditions where total travel time of the drivers is less but some drivers may not be in their quickest path. However, the linear subproblem of this formulation, which constrains the search direction, deviates from the objective surface, resulting in slow convergence of the Frank and Wolfe (FW) algorithm. In order to address this issue, Chen and Jayakrishnan^18^ studied a faster version of the FW algorithm, which employs a one-at-a-time flow upgrade strategy for each origin-destination (OD) pair. This new version can be mathematically represented as a linear program.

7 $$\begin{aligned} min \sum _{a \in A} t_{a} (z_{a}^n)y_{a}^{sd} \end{aligned}$$

subject to,

8 $$\begin{aligned}{} & {} \sum _{a \in O(n)} y_{a}^{sd} - \sum _{a' \in I(n)} y_{a'}^{sd} = {\left\{ \begin{array}{ll} q^{sd} &{} \quad if\, n=s\\ -q^{sd} &{}\quad if\, n=d, \quad \forall n \in N \\ 0 &{} \quad \text {otherwise} \end{array}\right. } \end{aligned}$$

9 $$\begin{aligned}{} & {} y_{a}^{sd} \ge 0, \quad \forall a \in A \end{aligned}$$

where $y_{a}^{sd}=$ flow from OD pair(s,d) being routed to link a; and $z_{a}^n=$ flow on link a resulting from the previous OD pair at iteration n.

In this version, the algorithm optimizes the user equilibrium by recalculating the shortest path and dividing flow based on the travel cost, as described in the previous version. The aim is to achieve a user equilibrium where no driver can improve their travel time by unilaterally switching to a different route. By iteratively adjusting the flow on each link, the algorithm eventually converges to a solution that satisfies the user equilibrium condition. This approach has been widely used in transportation planning and management to improve traffic flow efficiency and reduce congestion.

## Results

The proposed solution involves two steps, where road upgrade combinations are initially considered naively (CEM), followed by local area consideration to scale the algorithm (LDBM). To determine the quality of LDBM, we compare the total disruption resulting from scheduling all tasks at the same time under two scenarios. The first scenario involves no constraints on distance, while the second scenario only allows disruption from each task to fall within a specified distance. The difference in disruption between these two scenarios illustrates the quality of the approximation. Larger distances around each maintained road lead to better approximation quality, and our experimentation supports the selection of the optimal distance that yields a good approximation while minimizing the number of combinations that can be sufficiently tested. This section presents the experiments carried out using each algorithm to evaluate the performance of the proposed solution.

### Experiments

The real network data is taken from the Transportation Networks for Research Core Team^20^. The trip data is generated based on available nodes and the average capacity of the edges. Table 1 displays the data sets used to evaluate all the models. The created experiments and experiment results from CEM and LDBM are presented in the Tables 2, 3 and 4 respectively.

**Table 1:** Selected network data set.

City	Nodes	Edges
Sioux Falls	24	76
Chicago	12982	39018

### Comparison between CEM and LDBM

Our approach aims to determine the combinations of RPU that result in minimal disruption when scheduling RPU. To achieve this goal, we developed two algorithms: the combination evaluation model (CEM) and the Local Disruption-Based Model (LDBM). We used experimental data presented in Table 2 to evaluate both algorithms. The experiments were designed with a fixed number of trips. For the Chicago network, we conducted experiments with 500 trips for the small scale and 50,000 trips for the large scale. For the Sioux Falls network, we conducted three sets of experiments with 8, 37, and 300 trips. Road upgrade sites were selected to be dispersed throughout the road network. In Table 2, we introduced five road upgrade sites (A to E) into the Sioux Falls network and ten road upgrade sites (a to j) into the Chicago road network.

**Table 2:** Designed experiments to evaluate CEM and LDBM.

Experiment	City	Orders	Blocked edges
E1	Sioux Falls	8	[*D*]
E2	Sioux Falls	8	[*A*, *B*, *C*, *D*, *E*]
E3	Sioux Falls	37	[*D*]
E4	Sioux Falls	37	[*A*, *B*, *C*, *D*, *E*]
E5	Sioux Falls	300	[*D*]
E6	Sioux Falls	300	[*A*, *B*, *C*, *D*, *E*]
E7	Chicago	500	[*c*]
E8	Chicago	500	[*a*, *b*, *c*, *d*]
E9	Chicago	500	[*a*, *b*, *c*, *d*, *e*, *f*, *g*, *h*, *i*, *j*]
E10	Chicago	50,000	[*c*]
E11	Chicago	50,000	[*a*, *b*, *c*, *d*]
E12	Chicago	50,000	[*a*, *b*, *c*, *d*, *e*, *f*, *g*, *h*, *i*, *j*]

#### CEM

Table 3 presents the performance results of the CEM algorithm. The experiment pairs for each data set show that increasing the number of road upgrade sites has led to an exponential increase in runtime. Specifically, the runtime was ten times higher when RUS was increased from one to four. In the last test on the Chicago network, the algorithm that could solve for 50,000 OD pairs within 24 min could not resolve a schedule with ten road upgrade tasks within the given time limit.

As discussed in section “CEM”, the runtime exponentially increases with the number of tasks, as the algorithm considers all available combinations. Each combination reroutes all OD pairs and assumes that RUS affects all ODs. Considering the entire network and all available OD pairs increases the possibilities considered by the algorithm, making it infeasible within the given time. Based on these results, we introduced a local disruption-based model explained in section “LDBM”. The experiments were carried out within a time window of 12 h. Experiments that exceeded the time window to produce results are marked as unavailable (N/A).

**Table 3:** Experiment results for CEM.

Experiment	Execution time (s)	Initial UE	Minimum disruption	Schedule
E1	0.117	691.0	0.0	[*D*]
E2	0.147	691.0	56.0	[(*B*), (*A*, *C*, *D*, *E*)]
E3	0.116	1747.0	0.0	[*D*]
E4	0.178	1747.0	184.0	[(*A*, *D*), (*B*, *C*), (*E*)]
E5	0.164	20,412.0	0.0	[*D*]
E6	0.486	20,412.0	3756.0	[(*A*, *D*), (*B*, *C*), (*E*)]
E7	1.21	701,535.0	355	[*c*]
E8	2.3	701,535.0	453	[*a*, *b*, *c*, *d*]
E9	584	701,535.0	584	[*a*, *b*, *c*, *d*, *e*, *f*, *g*, *h*, *i*, *j*]
E10	97	7,331,744.0	10,990	[*c*]
E11	1440	7,331,744.0	12,269	[(*a*, *c*), (*b*), (*d*)]
E12	Timeout	7,331,744.0	NA	NA

#### LDBM

Table 4 displays the performance results of the LDBM algorithm. The comparison between Tables 3 and 4 illustrates that the runtime for all experiments improves with an increase in tasks. However, the last tests on the Chicago network indicate that the algorithm incurs higher costs when scheduling a few upgrade sites due to perimeter data calculations. In contrast, the benefits of local area calculation are achieved by increasing the number of tasks, as the algorithm searches for a locally feasible solution. In this way, the LDBM algorithm does not consider millions of orders, as in the CEM algorithm, which may result in an infeasible solution for the last experiment. Instead, the LDBM algorithm can find a solution within 1.3 s by identifying orders within the local area and evaluating the level of disruption caused. The LDBM algorithm eliminates most of the orders from consideration if they are not travelling through the local area. Moreover, it reduces the number of combinations to be evaluated by identifying RUS, which are independent.

**Table 4:** Experiment results for LDBM.

Experiment	Execution time (s)	Initial UE	Minimum disruption	Schedule
E1	2 ms	691.0	0.0	[*D*]
E2	0.175 s	691.0	56.0	[(*C*, *D*), (*A*, *B*, *E*)]
E3	6 ms	1747.0	78.0	[*D*]
E4	0.185 s	1747.0	433.0	[(*A*, *D*), (*B*, *E*), (*C*)]
E5	10 ms	20,412.0	2296.0	[*D*]
E6	0.424 s	20,412.0	5163.0	[(*A*), (*C*, *D*), (*B*, *E*)]
E7	0.5 s	701,535.0	416.0	[*c*]
E8	3.6 s	701,535.0	770	[*a*, *b*, *c*, *d*]
E9	1.2 s	701,535.0	905	[*a*, *b*, *c*, *d*, *e*, *f*, *g*, *h*, *i*, *j*]
E10	0.12 s	7,331,744.0	42,575	[*c*]
E11	0.55 s	7,331,744.0	44,924	[(*a*, *c*), (*b*), (*d*)]
E12	1.3 s	7,331,744.0	46,744	[(*d*, *e*), (*b*), (*g*), (*a*, *c*, *f*, *h*, *i*, *j*)]

Solid proof is required to demonstrate that the approximation algorithm produces solutions that are closer to the optimal solution. To evaluate the quality of the local disruption calculated by LDBM, a comparison is made against the global optimal solution. The global optimal is the maintenance schedule that minimises total user travel time, assuming a user equlibrium is reached for each set of maintenance activities occurring at the same time. The difference between these solutions serves as validation that the algorithm’s findings are indeed valid. The selection of a suitable radius to define the local area surrounding the road upgrade or maintenance site (RPU) plays a critical role in achieving a quality approximation within a shorter execution time. Table 5 presents the changes in local disruption calculation with varying radius, along with a comparison between global and local disruption. In the context of a road network, experiment data is analyzed to determine the appropriate radius for local disruption calculation. This choice is primarily based on the runtime and the proximity of the local disruption to the global disruption calculation. The results presented in Table 5 demonstrate the impact of changes in radius on local disruption calculation.

As the radius increases, the gap between local and global solutions becomes reduced. However, in real life, rerouting will not occur throughout the entire network, but will only consider the immediate neighborhood. Given this, the gap of 7000 for a radius of 3 when rerouting 50,000 origin-destination (OD) pairs is considered negligible.

**Table 5:** Experiment results for LDBM with increasing radius.

Experiment	Radius	Local disruption	Global disruption	Difference
Chicago-500 OD-1 RUS	3	416	355	61
Chicago-500 OD-1 RUS	15	416	355	61
Chicago-500 OD-1 RUS	70	355	355	0.0
Chicago-500 OD-4 RUS	16	755	453	92
Chicago-500 OD-4 RUS	50	453	453	0.0
Chicago-50,000 OD-4 RUS	6	12,269	19,276	7007.0

*OD* origin destination pairs, *RUS* road maintenance or upgrade sites.

## Discussion

The results section compares the naive scheduling approach with our local disruption-based approach to evaluate the scalability of our algorithm. The findings provide evidence that our algorithm shows promise in enhancing the scalability of scheduling multiple road upgrades by reducing the number of combinations that need to be evaluated. Experiment E12 in Table 2 could not be evaluated to determine the optimal schedule using the naive scheduling approach discussed in section due to the increase in possible combinations. In contrast, Table 3 and Table 4 demonstrate that the LDBM was able to find the optimal schedule for the given RPU sites within seconds. This approximation enhances the scalability of the solution by reducing the number of possible comparisons.

**Figure 6:**
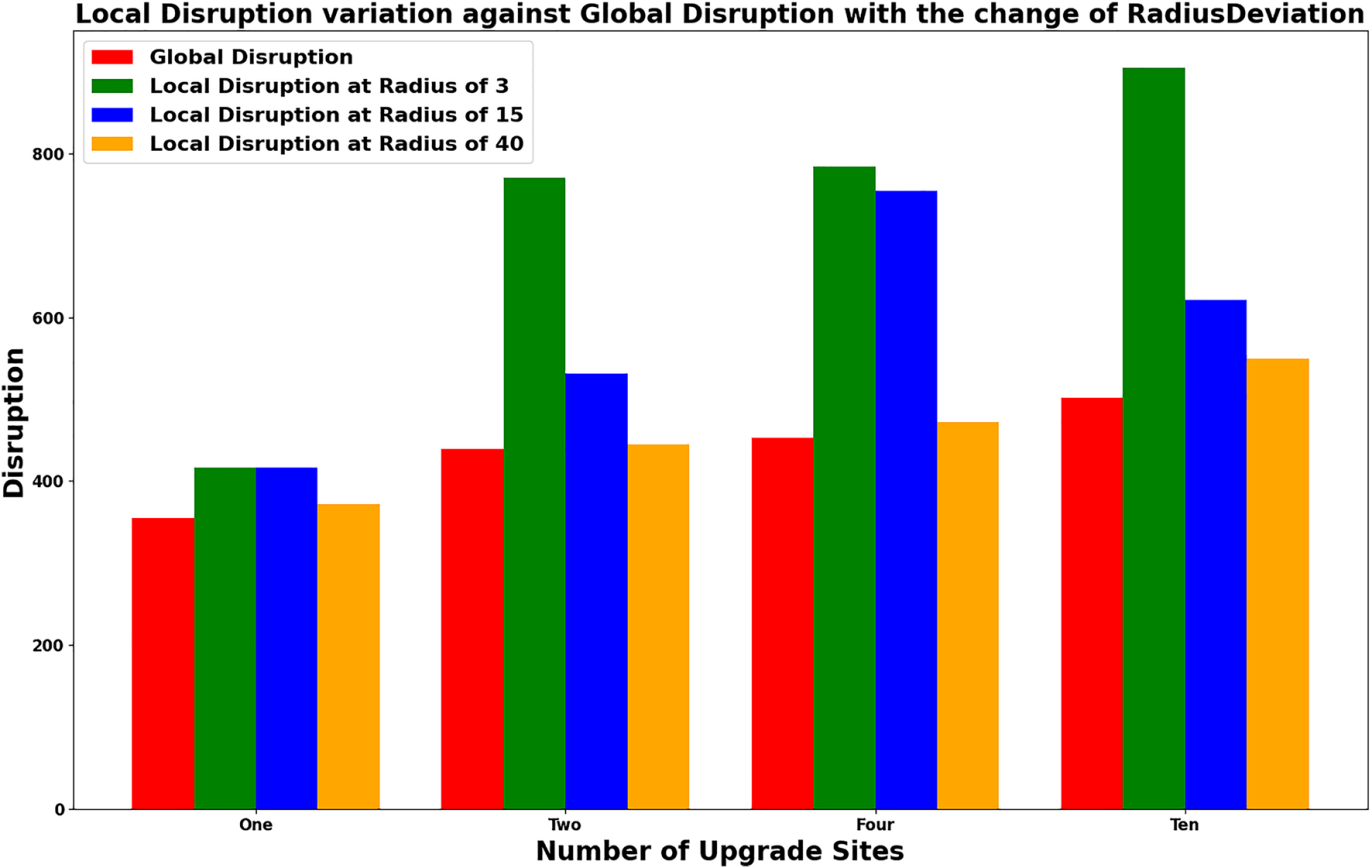
Deviation of local disruption with the change of radius.

The distinction between global and local disruption serves as evidence of the accuracy of our approach. We have demonstrated that by adjusting the radius of the local area, we can enhance the results to be more closely aligned with the global optimum. The hypothesis is supported by Fig. 6, where different radius values were selected and outcomes were assessed to demonstrate that a suitable radius produces a good approximation while effectively reducing the number of combinations that need to be evaluated.

**Figure 7:**
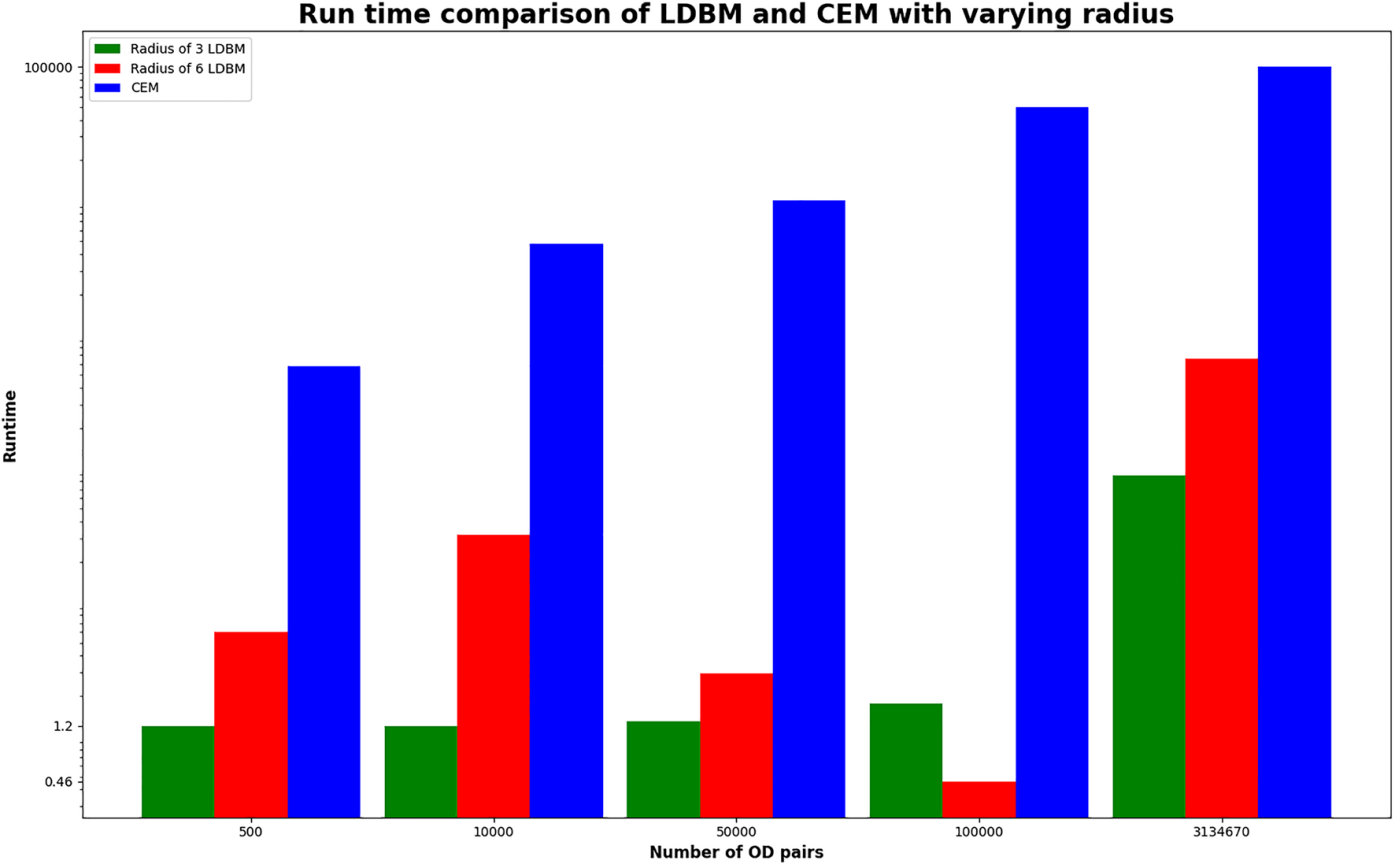
Evaluation of runtime improvement of LDBM compared to CEM.

Cities such as Chicago, USA, and Melbourne, Australia, have millions of road segments and trips that must be considered when developing road upgrades or maintenance schedules. The successful resolution of over fifty thousand trip data for the Chicago network demonstrates the practical applicability of our algorithm as a viable solution. Figure 7 shows how quickly our algorithm performs with an increasing number of OD pairs. For future work, we intend to enhance the scalability of our algorithm to schedule a greater number of road maintenance or upgrade sites while also accounting for resource constraints during scheduling.

## Conclusion

This study presents a comprehensive investigation into enhancing the scalability of scheduling multiple road upgrades through the utilization of a local disruption-based approach. The results of our empirical analysis, as outlined in the results section, establish a compelling case for the effectiveness and efficiency of our algorithm compared to the traditional naive scheduling approach. A key highlight of our approach lies in its adaptive nature, as demonstrated by the distinction between global and local disruption. By fine-tuning the radius of the local disruption area, we showcase the capacity to achieve results closely aligned with the global optimum, as verified through Fig. 6. This adaptability ensures that our algorithm can be tailored to specific scenarios, striking an optimal balance between accuracy and computational efficiency. While the current algorithm focuses on road segments with reduced capacity (due to maintenance), our approach could also be applied to assess increased capacity in existing or new road segments, thus supporting network upgrade planning. One limitation is that we do not consider other constraints when scheduling, such as resources and precedence. Hence, future work includes integrating the Resource-Constrained Project Scheduling Problem (RCPSP) into the current model. This enhancement will provide a more comprehensive and practical solution, further extending the applicability of our algorithm in addressing real-world road upgrade and maintenance challenges.

## Data Availability

The network datasets analysed during the current study are available in bstabler/TransportationNetworks repository^20^. The flow datasets generated during the current study are available in bhagyaj/Data repository^21^.
